# The co-treatment of rosuvastatin with dapagliflozin synergistically inhibited apoptosis via activating the PI3K/AKt/mTOR signaling pathway in myocardial ischemia/reperfusion injury rats

**DOI:** 10.1515/med-2021-0005

**Published:** 2020-12-11

**Authors:** Lei Gong, Xuyang Wang, Jinyu Pan, Mingjun Zhang, Dian Liu, Ming Liu, Li Li, Fengshuang An

**Affiliations:** The Key Laboratory of Cardiovascular Remodeling and Function Research, Chinese Ministry of Education, Chinese National Health Commission and Chinese Academy of Medical Sciences, The State and Shandong Province Joint Key Laboratory of Translational Cardiovascular Medicine, Department of Cardiology, Qilu Hospital, Cheeloo College of Medicine, Shandong University, No. 107 WenHuaXi Road, Jinan, Shandong 250012, China; The Second Affiliated Hospital of Xuzhou Medical University, No.32 MeiJian Road, Quanshan District, Xuzhou, Jiangsu 221000, China

**Keywords:** rosuvastatin, dapagliflozin, apoptosis, PI3K/AKt/mTOR, ischemia/reperfusion

## Abstract

**Objective:**

The purpose of the present study was to evaluate the role of co-treatment of rosuvastatin (RSV) and dapagliflozin (DGZ) preconditioning in myocardium ischemia/reperfusion (I/R) injury and to further investigate the underlying mechanism.

**Methods:**

Sprague-Dawley (SD) rats (*n* = 25) were divided into five groups randomly: (1) Sham, (2) I/R, (3) I/R + RSV (10 mg/kg), (4) IR + DGZ (1 mg/kg), and (5) I/R + RSV (10 mg/kg) + DGZ (1 mg/kg). The I/R model was induced with 30 min of left anterior descending occlusion followed by 120 min of reperfusion.

**Results:**

*In vivo* pretreatment with RSV and DGZ, respectively, showed a significant reduction of infarction size, a significant increase in the levels of left ventricular systolic pressure, and maximal rate increase in left ventricular pressure (+d*p*/d*t*_max_), decrease in the levels of left ventricular end-diastolic pressure (LVEDP), maximal rate of decrease of left ventricular pressure (−d*p*/d*t*_max_) and activity of cardiac enzymes of creatine kinase (CK), creatine kinase MB isoenzymes (CK-MB), and hyper-tensive cardiac troponin I compared with the I/R group. H9C2 cells were exposed to hypoxia/reoxygenation to simulate an I/R model. *In vitro* administration of 25 µM RSV and 50 µM DGZ significantly enhanced cell viability, upregulated the expression levels of p-PI3K, p-Akt, p-mTOR, and Bcl-2, whereas it downregulated cleaved-caspase3, Bax. TUNEL assay indicated that pretreatment with RSV and DGZ decreased the apoptosis of H9C2 cells.

**Conclusion:**

The combination of RSV and DGZ significantly enhances the cardioprotective effects compared with RSV or DGZ alone. RSV and DGZ have the potential cardioprotective effects against I/R injury by activating the PI3K/AKt/mTOR signaling pathway.

## 1 Introduction

It has been reported that acute myocardial infarction contributes to high rates of morbidity and mortality in the past few decades in China [1]. In recent 20 years, the prompt myocardial revascularization can recover the myocardium blood flow and reduce ischemia-induced injury [2]. However, it may inevitably cause contractile dysfunction, severe arrhythmias, extremely to death, the complicated pathophysiological process of which was referred to as myocardial ischemia/reperfusion (I/R) injury [3]. A number of previous studies have demonstrated that the underlying mechanism of myocardial I/R injury was relevantly related to apoptosis [4,5].

Apoptosis is a complex regulated pathological process in the programed cell death, playing a vital role during the myocardial I/R injury. Numerous studies have confirmed that apoptosis was stimulated to reduce the myocardium cell survival via phosphatidylinositol-3-kinase (PI3K)/protein kinase B (AKt)/mechanistic target of the rapamycin (mTOR) signaling pathway in the acute myocardial I/R injury [6,7]. Unfortunately, there are few effective therapy strategies for preventing the process of myocardial I/R injury. Therefore, in clinical practice, it is of great importance and necessity to explore and resolve the intricate I/R-associated phenomenon.

The statins, 3-hydroxy-3-methylglutaryl-CoA (HMG-CoA) reductase inhibitors, are effective in the treatment of dyslipidemia [8]. In recent years, previous studies have demonstrated that rosuvastatin (RSV) can decrease cardiovascular morbidity and mortality, and reduce myocardial I/R injury [9]. Dapagliflozin (DGZ), the sodium glucose co-transporter 2 inhibitors (SGLT2I), is a novel class of anti-diabetic drugs, which results in inhibiting glucose reabsorption and excretion of glucose into the urine [10]. A recent trial showed that DGZ had a greater efficacy on cardioprotection than vildagliptin in rats with cardiac I/R injury [11].

Although the cardioprotection of RSV or DGZ on ischemic reperfusion myocardium has been demonstrated in several experiments [9–11], the combined effects of RSV and DGZ on the cardiovascular system have not been reported yet. This study aimed to investigate whether the preconditioning with the combination of RSV and DGZ is superior to the RSV or DGZ alone in the treatment of cardiac I/R injury, and if so, whether the PI3K/AKt/mTOR signaling pathway still plays a key role in it.

## 2 Materials and methods

### 2.1 Animals and drugs

Eight-week-old male Sprague-Dawley (SD) rats were obtained from HFK Bioscience Company (Beijing, China). All rats were fed regular chow and maintained under standard lighting (12:12 h, day–night rhythm), temperature (20–22°C), and humidity (50–60%). After 1 week of acclimation, all the rats were randomly divided into the following five groups (*n* = 5 per group): Sham (S) group, I/R group, I/R + rosuvastatin (RSV, Crestor, Batch No. 115722) group, I/R + dapagliflozin (DGZ, Forxiga, Batch No. JH2197) group, and I/R + RSV + DGZ group. The S group and I/R group were given normal diet. The I/R + RSV group was administered RSV dissolved in distilled water via oral gavage at a dose of 10 mg/kg/day for 7 days continuously [12,13], the I/R + DGZ group received 1 mg/kg/day DGZ dissolved in distilled water by oral gavage for 7 days continuously [11], and the I/R + RSV + DGZ group received RSV 10 mg/kg/day and DGZ 1 mg/kg/day simultaneously via oral gavage for 7 days consecutively. The animals were forbidden to feed overnight before experiment, but were given free access to water. The experimental protocols in the present study were approved by the Animal Ethics Committee of Shandong University. All experimental procedures were carried out in accordance with experimental ethics.

### 2.2 Myocardial ischemia/reperfusion procedure

All rats were anesthetized and tracheotomy intubated artificially. The measurement catheter was used for hemodynamic monitoring, and to record the left ventricular systolic pressure (LVSP), left ventricular end-diastolic pressure (LVEDP), and maximal rate of increase and decrease in left ventricular pressure (±d*p*/d*t*_max_). A 5-0 silk was placed around the left anterior descending (LAD) artery for ligating. LAD was occluded for 30 min, reperfusion was initiated by releasing the ligature for 120 min [14]. The S group only underwent a similar surgical procedure without ligating. All rats were sacrificed at the end of the experiment; the blood and heart were collected timely.

### 2.3 Measurement of serum enzymes in cardiac tissues

Blood samples were obtained from the apex following reperfusion for 120 min and were centrifuged at 3,000 rpm for 10 min at 4°C. The serum levels of creatine kinase (CK), creatine kinase MB isoenzymes (CK-MB), and hyper-tensive cardiac troponin I (hs-cTNI) were measured by automatic analysis apparatus (Beckman Coulter AU680, USA; Simens ADVIA Centaur CP, Germany).

### 2.4 Measurement of myocardial infarct size

After 120 min of perfusion, all the rats were sacrificed, the hearts were collected, and Evans blue/triphenyltetrazolium chloride (TTC) staining was performed to assess the area at risk (AAR) and infarct size as previously described [15]. Briefly, after staining, white and red parts represented the infarct size and ischemic but viable tissue, respectively. Evans blue-stained areas indicated non-ischemic area. White plus red part was AAR.

### 2.5 Cell cultures and treatments

H9C2 cardiomyocytes were purchased from the Type Culture Collection of the Chinese Academy of Sciences (Shanghai, China). H9C2 cells were cultured and incubated for 48 h; five replicates were made for each group. The cells were divided into control group (C group) and five hypoxia/reoxygenation model groups (H/R group). The C group was incubated in 4.5 g/L glucose Dulbecco’s modified Eagle’s medium (DMEM; Gibco, China) supplied with 10% FBS. Five H/R groups were transferred, respectively, into sugar and serum-free DMEM containing RSV (purity > 99%, Meilunbio, China) and DGZ (purity > 99%, MCE, USA) under five various concentrations of 6.25, 12.5, 25, 50, and 100 µM to select an optimal protection concentration and incubated in 1% O_2_, 5% CO_2_, and 94% N_2_ at 37°C for 2 h then transferred into 4.5 g/L glucose DMEM supplied with 10% FBS and incubated in 5% CO_2_ combined with 95% room air at 37°C for 6 h. Cell viability assay was then analyzed through Cell Counting-Kit 8 (CCK-8; Beyotime, China). The results were expressed as the relative percentage of the C group, which was considered 100% viable.

H9C2 cells were cultured and divided into five groups randomly as follows: Control(C) group, H/R group, H/R + RSV group, H/R + DGZ group, and H/R + RSV + DGZ group. Sugar and serum-free DMEM in the cubator with the optimal protective concentration of 25 µM RSV and 50 µM DGZ, respectively.

### 2.6 Detection of extracellular lactate dehydrogenase (LDH) activity

The activity of LDH in the supernatant of H9C2 cells with different treatment groups was measured using LDH assay kit (Beyotime, China) following the specification to evaluate the presence of necrotic cell death. The levels of LDH were measured through the absorbance values at a wavelength of 490 nm using Spectra Max Plus apparatus (Spectra Max Plus 384; Molecular Devices, USA).

### 2.7 Western-blot analysis

The total cardiomyocyte cellular protein was loaded onto an 8–12% sodium dodecyl sulfate-polyacrylamide gels, and then transferred to polyvinylidene fluoride membranes (Millipore, USA). The membranes were sealed and incubated with particular primary antibodies targeting phosphorylated phosphatidylinositol 3-kinase (p-PI3K) (rabbit polyclonal, ab70912; Abcam), phosphatidylinositol 3-kinase (PI3K) (rabbit polyclonal, ab191606; Abcam), phosphorylated protein kinase B (p-AKt) (rabbit polyclonal, ab38449; Abcam), protein kinase B (AKt) (rabbit polyclonal, ab64148; Abcam), phosphorylated mammalian target of rapamycin (p-mTOR) (rabbit polyclonal, ab226957; Abcam), mammalian target of rapamycin (mTOR) (rabbit polyclonal, ab32028; Abcam), B-cell lymphoma 2 (Bcl-2)(rabbit polyclonal, ab32124; Abcam), Bcl-2 associated X protein (Bax) (rabbit polyclonal, ab32503; Abcam), caspase3 (rabbit polyclonal, ab13847; Abcam), cleaved-caspase3 (rabbit polyclonal, ab2302; Abcam), and β-actin (rabbit polyclonal, ab8227; Abcam) overnight at 4°C. The protein expression of the membranes was visualized using chemiluminescent kits (Millipore, USA) via ultrasensitive chemiluminescence imager (GE Amersham Imager 600, USA). The gray-scale values of the target bands were analyzed by Image J.

### 2.8 TUNEL assay

The DNA fragmentation of apoptotic cells was detected by TUNEL staining. According to the manufacturer’s instructions, cells were permeabilized with 0.1% TritonX-100 for 5 min at 4°C. Apoptotic H9C2 cells were performed using the *In situ* Cell Death Detection Kit, TMR red (Roche, 12156792910, Germany) followed instructions and 4′,6-diamidino-2-phenylindole (DAPI, Beyotime, China) for 5 min, subsequently, observed under a microscope (Nikon, Japan). The index of cell apoptosis was calculated as the percentage of apoptotic nuclei/total nuclei.

### 2.9 Statistical analysis

Data were expressed as mean ± standard deviation (\bar{x} ± SD). SPSS version 20.0 (IBM SPSS Statistics, USA) was used for statistical analysis. Differences were evaluated using one-way analysis of variance among the groups. *P* < 0.05 was considered to be statistically significant.

## 3 Results

### 3.1 Hemodynamics in myocardium I/R injury rats

**Figure 1:**
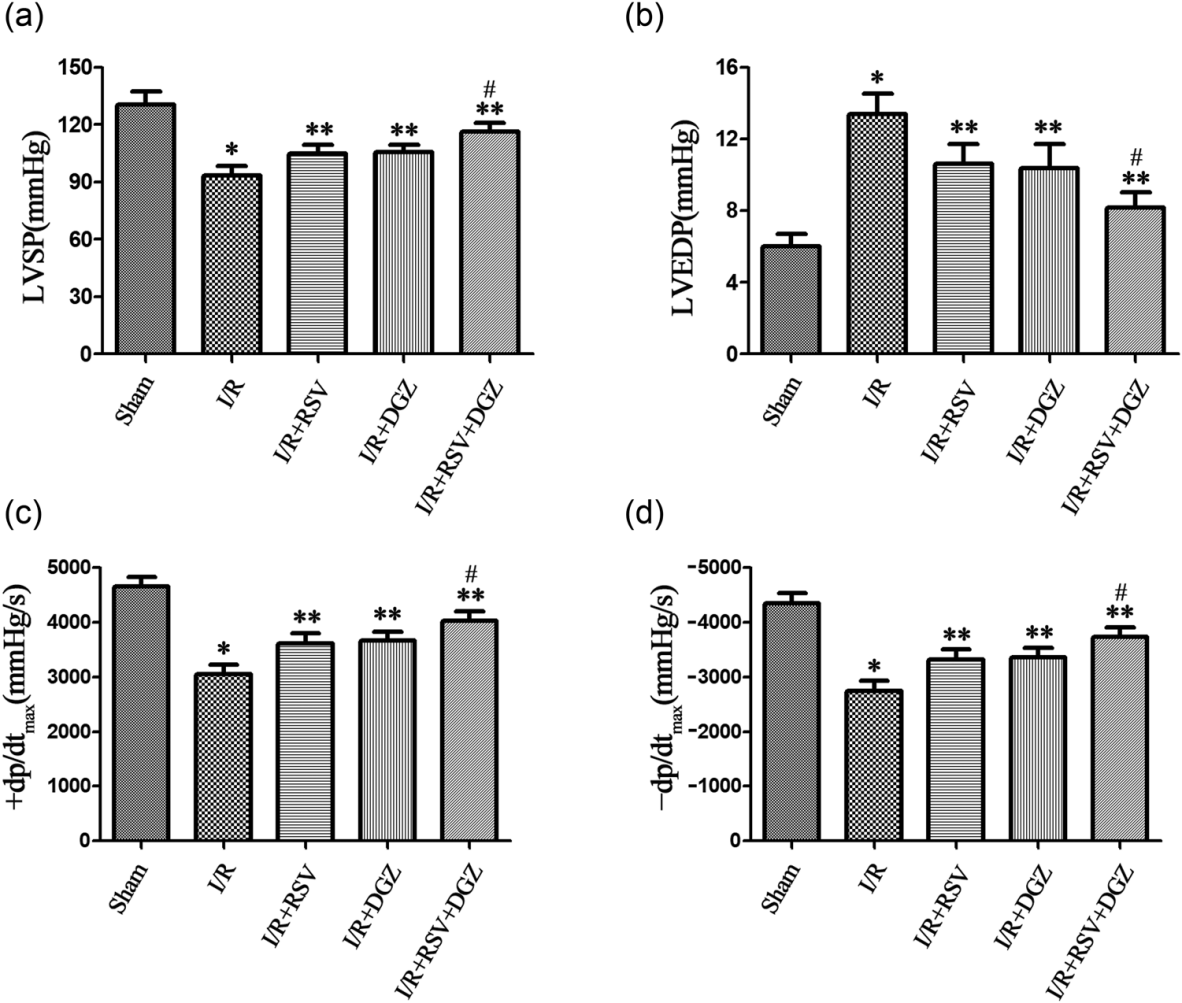
Hemodynamics in rats subjected to 30 min ischemia and 120 min of reperfusion for each group (mean ± SD, *n* = 5). *p < 0.001 compared with Sham; **p < 0.05 compared with I/R; ^#^ *p* < 0.05 compared with I/R + RSV or I/R + DGZ. LVSP: left ventricular systolic pressure; LVEDP: left ventricular end-diastolic pressure; +d*p*/d*t*_max_: maximal rate of increase left ventricular pressure; −d*p*/d*t*_max_: maximal rate of decrease left ventricular pressure. Sham: sham operated group; I/R: ischemia/reperfusion group; I/R + RSV: ischemia/reperfusion + rosuvastatin group; I/R + DGZ: ischemia/reperfusion + dapagliflozin group; I/R + RSV + DGZ: ischemia/reperfusion + rosuvastatin + dapagliflozin group.

SD rats’ hearts were subject to 30 min ischemia, and 120 min reperfusion showed significant decrease in mechanical function (Figure 1). The levels of LVSP and +d*p*/d*t*_max_ were significantly decreased, while LVEDP and −d*p*/d*t*_max_ were significantly increased in the I/R group compared to those of the sham group (*p* < 0.001). Administration of RSV or DGZ, respectively, significantly increased the levels of LVSP, +d*p*/d*t*_max_ and decreased the levels of LVEDP, −d*p*/d*t*_max_ in the I/R + RSV or I/R + DGZ group compared to that of the I/R group (*p* < 0.05). When RSV and DGZ were administered simultaneously, the levels of LVSP, +d*p*/d*t*_max_ significantly increased and the levels of LVEDP, −d*p*/d*t*_max_ decreased, superior to RSV or DGZ, respectively (*p* < 0.05).

### 3.2 Effects of RSV and DGZ on the intracellular cardiac enzyme levels in SD rats

**Figure 2:**
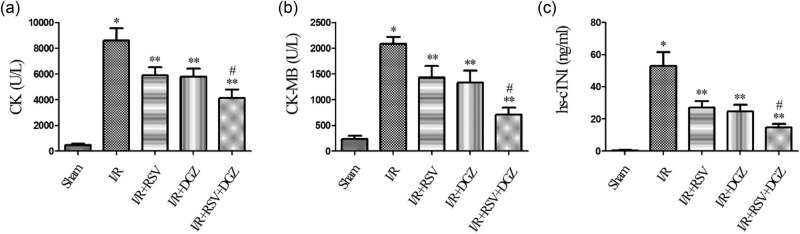
Comparison of the serum CK, CK-MB, and hs-cTNI levels across various experimental groups of SD rats (mean ± SD, *n* = 5). *p < 0.001 compared with Sham; **p < 0.05 compared with I/R; ^#^ *p* < 0.05 compared with I/R + RSV or I/R + DGZ. Sham: sham operated group; I/R: ischemia/reperfusion group; I/R + RSV: ischemia/reperfusion + rosuvastatin group; I/R + DGZ: ischemia/reperfusion + dapagliflozin group; I/R + RSV + DGZ: ischemia/reperfusion + rosuvastatin + dapagliflozin group.

Compared with the S group, the levels of CK, CK-MB, and hs-cTNI in other four groups were increased (*p* < 0.001). The levels of CK, CK-MB, and hs-cTNI were reduced when pretreated with RSV and DGZ compared to the I/R group (*p* < 0.05). When pretreated with RSV and DGZ simultaneously, the levels of CK, CK-MB, and hs-cTNI significantly reduced, superior to RSV or DGZ, respectively (*p* < 0.05) (Figure 2).

### 3.3 RSV and DGZ decrease I/R-induced myocardial infarct size in SD rats

Table 1 shows the results of heart sections in five groups stained with Evans Blue/TTC. There was no significant difference in the AAR/LV (%) between all hearts when exposed to I/R treatments. The infarct sizes (% of AAR) in I/R + RSV and I/R + DGZ groups were significantly decreased compared with the I/R group (*p* < 0.01), but inferior to the co-treatment of RSV and DGZ groups (*p* < 0.01).

**Table 1:** Effect of the RSV and DGZ on AAR/LV and infarct size in hearts

Group	AAR/LV (%)	Infarct size/AAR (%)
Sham	—	—
I/R	45.70 ± 3.76	34.96 ± 3.08
I/R + RSV	45.38 ± 3.37	27.26 ± 2.80^*^
I/R + DGZ	45.12 ± 3.05	26.48 ± 2.78^*^
I/R + RSV + DGZ	44.58 ± 3.21	22.46 ± 1.90^*#^

^*^ *p* < 0.01, compared with the H/R group; ^#^ *p* < 0.01, compared with the I/R + RSV or I/R + DGZ group. Sham: sham operated group; I/R: ischemia/reperfusion group; I/R + RSV: ischemia/reperfusion + rosuvastatin group; I/R + DGZ: ischemia/reperfusion + dapagliflozin group; I/R + RSV + DGZ: ischemia/reperfusion + rosuvastatin + dapagliflozin group.

### 3.4 RSV and DGZ improve the viability of H9C2 cells induced by H/R

**Figure 3:**
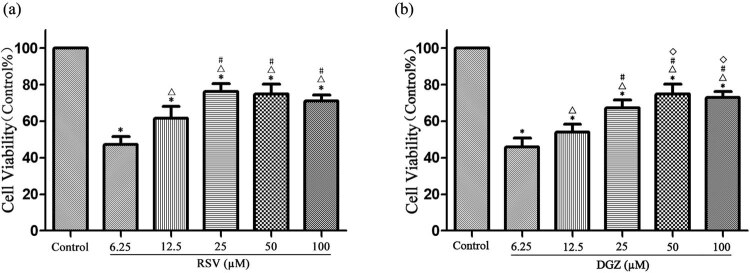
Comparison of the cell viability of various concentrations rosuvastatin and dapagliflozin pretreatment the H/R-induced H9C2 cells using a CCK-8 assay (mean ± SD, *n* = 6). *p < 0.001 compared with the control group; # *p* < 0.01 compared with the 6.25 µM group; △ *p* < 0.01 compared with the 12.5 µM group; ◇ *p* < 0.01 compared with the 25 µM group. RSV: rosuvastatin; DGZ: dapagliflozin. Control: control group; 6.25 µM: the concentration of 6.25 µM dapagliflozin pretreatment the hypoxia/reoxygenation induced H9C2 cells group; 12.5 µM: the concentration of 12.5 µM dapagliflozin pretreatment the hypoxia/reoxygenation induced H9C2 cell group; 25 µM: the concentration of 25 µM dapagliflozin pretreatment the hypoxia/reoxygenation induced H9C2 cell group; 50 µM: the concentration of 50 µM dapagliflozin pretreatment the hypoxia/reoxygenation induced H9C2 cell group; 100 µM: the concentration of 100 µM dapagliflozin pretreatment the hypoxia/reoxygenation induced H9C2 cell group.

The viability of H9C2 cells was significantly decreased in H/R groups compared to the control group (*p* < 0.001). Cell survival rates were significantly improved by various concentrations of RSV and DGZ pretreatment, respectively. RSV or DGZ at concentrations ranging from 6.25 to 100 µM significantly increased cell viability in a dose-dependent manner. The optimal cardioprotection concentration of RSV and DGZ was selected in the five concentrations. Consistent with the above finding, 25 µM RSV and 50 µM DGZ offered the best protection in the H/R-induced H9C2 cells, respectively (Figure 3).

### 3.5 LDH release was reduced by RSV and DGZ in H/R-induced H9C2 cells

LDH release was a substantial increase in culture media occurring as a result of H/R compared with the control (*p* < 0.001) (Table 2). However, following exposure to RSV or DGZ, the levels of LDH were extraordinarily lower than that of H/R. In the RSV and DGZ groups, the release of LDH was significantly decreased compared with the RSV or DGZ group (*p* < 0.01). Both RSV and DGZ significantly reduced LDH levels but there was no significant difference between the two groups (*p* = 0.36).

**Table 2:** Comparison of the activity of LDH in the five groups (mean ± SD, *n* = 6)

Group	Activity of LDH (control %)
Control	100
H/R	409.69 ± 50.39^*^
H/R + RSV	353.55 ± 51.42^*#^
H/R + DGZ	332.78 ± 40.18^*#^
H/R + RSV + DGZ	257.81 ± 21.81^*#△^

^*^ *p* < 0.001, compared with the control group; ^#^ *p* < 0.01, compared with the H/R group; △*p* < 0.01, compared with the H/R + RSV or H/R + DGZ group. H/R: hypoxia/reoxygenation group; H/R + RSV: hypoxia/reoxygenation + rosuvastatin group; H/R + DGZ: hypoxia/reoxygenation + dapagliflozin group; H/R + RSV + DGZ: hypoxia/reoxygenation + rosuvastatin + dapagliflozin group.

### 3.6 Effects of RSV and DGZ on apoptosis-related protein and PI3K/AKt/mTOR pathway during H/R in H9C2 cells

**Figure 4:**
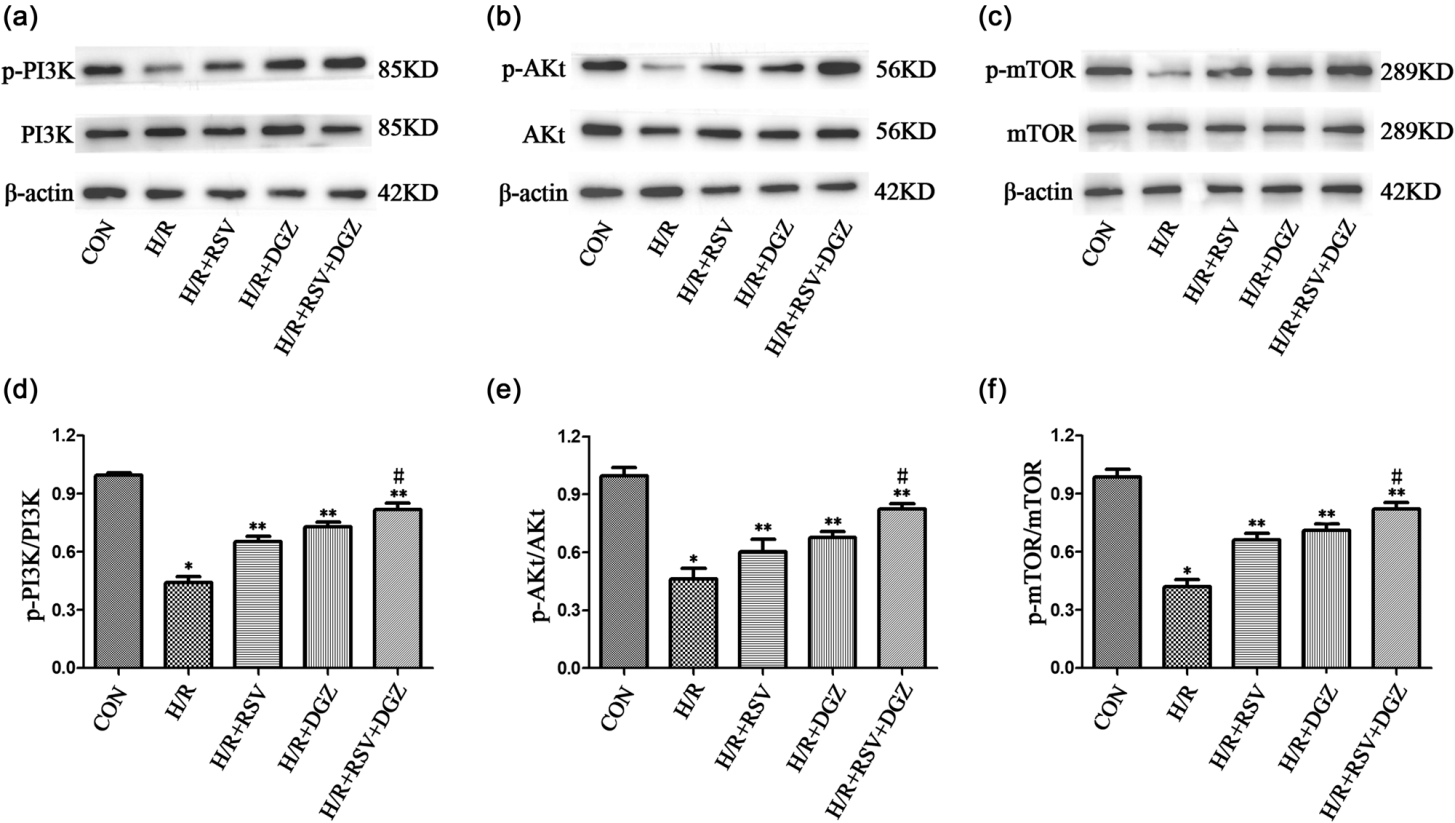
The effects of rosuvastatin and dapagliflozin on the PI3K/AKt/mTOR signaling pathway in H/R-induced H9C2 cells (mean ± SD, *n* = 3). *p < 0.001 compared with CON; **p < 0.05 compared with H/R; #*p* < 0.05 compared with H/R + RSV or H/R + DGZ. CON: control group; H/R: hypoxia/reoxygenation group; H/R + RSV: hypoxia/reoxygenation + rosuvastatingroup; H/R + DGZ: hypoxia/reoxygenation + dapagliflozin group; H/R + RSV + DGZ: hypoxia/reoxygenation + rosuvastatin + dapagliflozin group. p-: phosphorylated; PI3K: phosphatidylinositol-3-kinase; AKt: protein kinase B; mTOR: mechanistic target of rapamycin.

Apoptosis is mediated by the PI3K/AKt/mTOR signaling pathway. The expression of p-PI3K, PI3K, p-AKt, AKt, p-mTOR, and mTOR were measured using western-blot analysis in different groups (Figure 4a–c). The data showed that the expression of p-PI3K, p-AKt, and p-mTOR were significantly decreased in the H/R group (*p* < 0.001) (Figure 4d–f). The levels of p-PI3K, p-AKt, and p-mTOR were significantly increased when pretreated with RSV and DGZ, respectively, or simultaneously compared to the H/R group (*p* < 0.05) (Figure 4d–f). Immunoblotting results illustrated that the PI3K/AKt/mTOR pathways were activated by RSV and DGZ in H/R-induced H9C2 cells.

**Figure 5:**
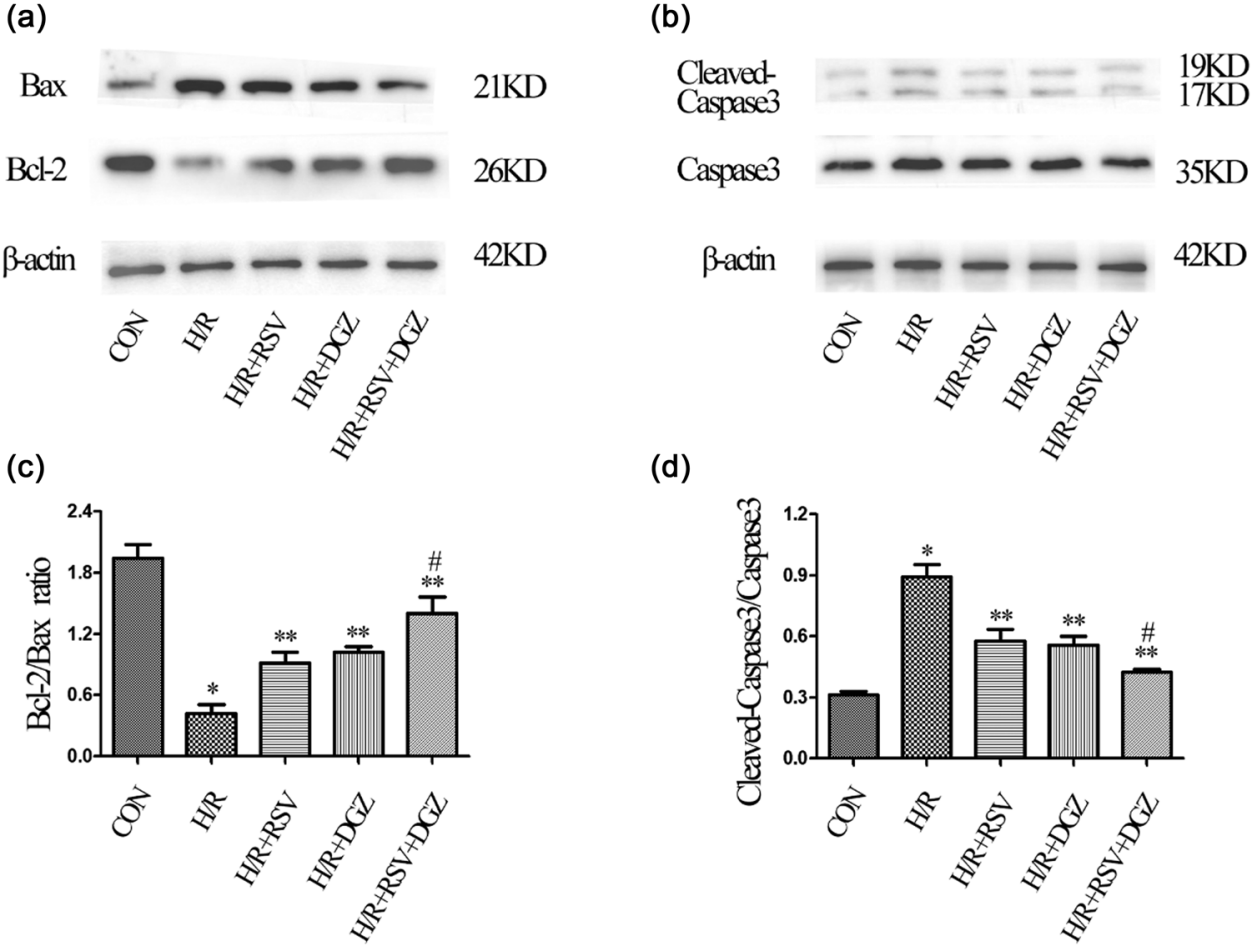
The expression levels of myocardial Bcl-2, Bax, cleaved-caspase3, and caspase3, the quantification of the Bcl-2/Bax ratio and cleaved-caspase3/caspase3 in the various groups (mean ± SD, *n* = 3). *p < 0.001 compared with CON; **p < 0.05 compared with H/R; ^#^ *p* < 0.05 compared with H/R + RSV or H/R + DGZ. CON: control group; H/R: hypoxia/reoxygenation group; H/R + RSV: hypoxia/reoxygenation + rosuvastatin group; H/R + DGZ: hypoxia/reoxygenation + dapagliflozin group; H/R + RSV + DGZ: hypoxia/reoxygenation + rosuvastatin + dapagliflozin group. Bcl-2: B-cell lymphoma 2; Bax: Bcl-2-associated X protein.

The activity of cleaved-caspase3, Bcl-2, and Bax was explored to confirm the characteristic features of apoptosis in H/R-indicated H9C2 cells by western-blot analysis. The analysis indicated the expression changes of Bcl-2 and Bax, two proteins associated with apoptosis. H/R downregulated the expression of Bcl-2 and upregulated Bax expression (*p* < 0.001) (Figure 5a). Pretreatment with RSV and DGZ resulted in the upregulation of Bcl-2 and downregulation of Bax (*p* < 0.05) (Figure 5c). The activity of cleaved-caspase3 was significantly enhanced in H9C2 cells following treatment of H/R (*p* < 0.001). However, when pretreatment of RSV and DGZ was performed, the activity of cleaved-caspase3 was significantly inhibited (*p* < 0.05) (Figure 5b). Western-blot analysis also demonstrated that H/R upregulated the expression of caspase-3, while RSV and DGZ treatment suppressed this expression (*p* < 0.05) (Figure 5d).

### 3.7 Cell apoptosis was attenuated by RSV and DGZ in H/R-induced H9C2 cells

**Figure 6:**
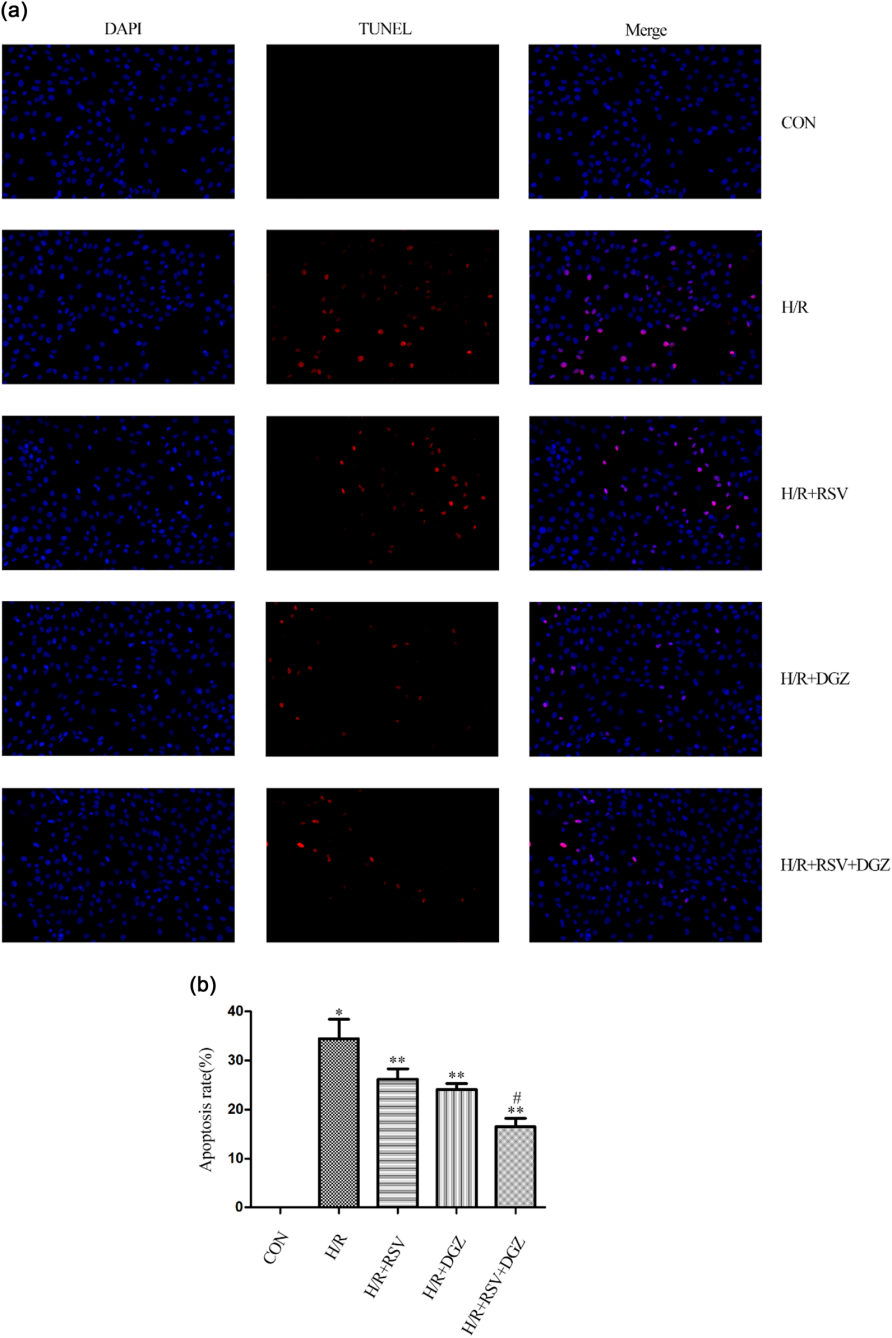
Protection effect of RSV and DGZ against H/R-induced H9C2 cells. Cell apoptosis was assessed using TUNEL staining (mean ± SD, *n* = 3) (magnification, ×200). (a) Representative images of TUNEL. Apoptotic nuclei were stained with TUNEL (red) and total nuclei staining with DAPI (blue). (b) Bar diagram showing the relative proportion of TUNEL-positive cells. *p < 0.001 compared with CON; **p < 0.05 compared with H/R; ^#^ *p* < 0.05 compared with H/R + RSV or H/R + DGZ. CON: control group; H/R: hypoxia/reoxygenation group; H/R + RSV: hypoxia/reoxygenation + rosuvastatin group; H/R + DGZ: hypoxia/reoxygenation + dapagliflozin group; H/R + RSV + DGZ: hypoxia/reoxygenation + rosuvastatin + dapagliflozin group.

TUNEL assay demonstrated that cells with red nuclei were considered apoptotic. Few cells with nuclei staining red were observed in the control group. H/R could cause apparent apoptosis as compared with the control group (*p* < 0.001) (Figure 6a). However, the cell apoptosis in H/R-induced H9C2 cells was significantly attenuated by RSV or DGZ pretreatment, respectively, especially after the pretreatment of combination with RSV and DGZ. This protection on H9C2 cells was evaluated by TUNEL-DAPI co-staining.

## 4 Discussion

In previous studies, SD rats were widely utilized to construct the myocardial I/R injury models and demonstrated that myocardial I/R injury is the major cause of cardiomyocyte apoptosis [16]. It has been found that the myocardial I/R injury can lead to severe myocardial necrosis and apoptosis, eventually myocardial infarction and cardiac dysfunction [17]. It is suggested that the PI3K/AKt/mTOR signaling pathway is involved in the apoptotic process of cardiomyocytes [18]. The pretreatment of RSV or DGZ has been shown to protect the myocardium against I/R injury and reduce the release of cardiac enzymes and apoptosis [9–11]. Although the cardioprotection of RSV or DGZ on myocardial I/R injury has been demonstrated in several experiments, the combined effects of RSV and DGZ on the cardiovascular system have not been reported yet. Therefore, in this study, we explore the regulation of myocardial I/R injury and the underlying molecular mechanism of the PI3K/AKt/mTOR signaling pathway in H9C2 cells under the effect of RSV and DGZ alone or synergistic.

Two major findings were discovered in the present study. RSV and DGZ preconditioning could significantly alleviate myocardial injury, reduce the release of cardiac enzymes, and decrease cardiomyocyte apoptosis. The cardioprotective effects of RSV and DGZ through activating the PI3K/AKt/mTOR signaling pathway were confirmed.

The myocardial I/R injury models presently showed various hemodynamics parameters such as heart rate, LVSP, LVEDP, +d*p*/d*t*_max_, and −d*p*/d*t*_max_. LVEDP is an index reflecting the left ventricular preload, which depends on the returned blood volume before ventricular systole and cardiac ejection function [19]. LVEDP and −d*p*/d*t*_max_ will be increased, LVSP and +d*p*/d*t*_max_ will be decreased when myocardial diastolic and systolic functions are inhibited [20]. In this experiment, compared with the S group, LVEDP and −d*p*/d*t*_max_ in the I/R group significantly increased, while LVSP and +d*p*/d*t*_max_ decreased, indicating that the myocardial systolic and diastolic functions were impaired after myocardial infarction. When pretreated with RSV or DGZ alone, left ventricular systolic and diastolic dysfunctions were promoted, indicating that RSV or DGZ can reduce LVEDP, improve LVSP, increase +d*p*/d*t*_max_, and decrease −d*p*/d*t*_max_. When pretreated with RSV combined with DGZ, the myocardial systolic and diastolic functions were significantly improved than RSV or DGZ alone.

The serum level of hs-cTNI is widely used in the assessment of myocardium injury and expressed almost exclusively in the heart [21]. CK and CK-MB are relatively highly sensitive and specific in predicting the acute myocardium infarction [22]. Particularly, hs-cTNI is the preferred biomarker for the evaluation of acute myocardial injury [23]. In the present study, it was demonstrated that the release of cardiac enzyme in the I/R group was much higher than other groups. In contrast, the administration of RSV or DGZ alone pretreatment was able to produce cardioprotective effects on myocardium against infarct size or biochemical parameters, and the RSV group was slightly inferior to the DGZ group, but there had been no significant difference. Pretreatment with RSV and DGZ synergistically significantly improved cardiac function than RSV or DGZ alone.

Cell viability was examined by the CCK-8 assay, and cell apoptosis was assessed by the TUNEL assay. The CCK8 assay indicated that the cell viability was obviously reduced after H/R. The cell viability in various groups was significantly different. Apoptosis, a form of programmed cell death, is an important mechanism for H9C2 cell H/R injury. Previous studies have shown that RSV or DGZ protected myocardium against H/R injury via inhibiting cardiomyocyte apoptosis [11,24]. In this study, the level of myocardial cell apoptosis was evaluated by TUNEL staining, a sensitive index of evaluating apoptosis [25]. It was found that the proportion of myocardial cell apoptosis was much higher in the H/R group than that in the C group, while pretreatment with RSV and DGZ could reduce myocardial cell apoptosis after myocardium H/R. Our study verified that RSV and DGZ inhibit apoptotic cardiomyocytes in the H/R group.

The result also showed that the apoptotic cells were significantly reduced after RSV and DGZ pretreatment. At the same time, the LDH levels of the supernatant of H9C2 cells in the H/R group decreased than other groups. RSV and DGZ pretreatment could attenuate H/R-induced H9C2 cellular damage, indicated by decreased apoptosis and LDH release.

It was reported that the reduction of apoptosis depended on the activation of the PI3K/AKt/mTOR signaling pathway [26]. In our study, the western-blot test was used to detect the protein expression of those key components in the PI3K/AKt/mTOR signaling pathway. Results suggested that the PI3K/AKt/mTOR pathway was activated by RSV and DGZ alone or synergistical pretreatment, the phosphorylated levels of PI3K, AKt, and mTOR were all increased, which in turn suppressed the pro-apoptotic activity of apoptosis [27].

Bcl-2 family proteins were known to exert a vital effect in the regulation of apoptosis; it includes both anti-apoptotic protein Bcl-2 and pro-apoptotic protein Bax [28]. The Bcl-2/Bax ratio has been emerged as a marker representing the extent of apoptosis. In the present study, the expression of Bcl-2 and Bax in the protein level by western-blot test was investigated. The Bcl-2/Bax ratio in the RSV or DGZ pretreatment group was significantly increased compared with that in the H/R group, but much lower than that in the RSV + DGZ group. This indicates that RSV and DGZ inhibited apoptosis through upregulating the anti-apoptosis protein expression of Bcl-2 and downregulating the pro-apoptosis protein Bax.

Caspase-3 is an essential protease, which is considered to play an important role in the apoptotic cascade reaction. It becomes cleaved caspase-3 when activated in the early phase of apoptosis [29]. Consistent with the previous study, we observed that the expression of cleaved caspase-3 of the H/R group was much higher than the control group. However, pretreatment with RSV and DGZ in the H/R group decreased cleaved caspase-3, showing that RSV and DGZ play a protecting role.

When pretreated with RSV and DGZ in the H/R group, the levels of pro-apoptotic protein cleaved caspase-3 and Bax were downregulated, followed by the upregulations of anti-apoptotic protein Bcl-2 and Bcl-2/Bax ratio.

Therefore, these results confirmed that the antiapoptotic effect of the RSV and DGZ pretreatment should be responsible for the cardioprotection against H/R injury. Numerous studies have shown that activating the PI3K/AKt/mTOR pathway exerts beneficial effects on ischemic hearts [30]. Results suggested that the PI3K/AKt/mTOR pathway was activated by RSV and DGZ alone or synergistical pretreatment, the phosphorylated levels of PI3K, AKt, and mTOR were all increased, which in turn suppressed the pro-apoptotic activity of apoptosis [27]. In our study, the western-blot test was used to detect the protein expression of those key components in the PI3K/AKt/mTOR signaling pathway. In the PI3K/AKt/mTOR signaling pathway, mTOR is in the downstream of the PI3K/AKt pathway and its activity is mainly regulated by the PI3K/AKt signaling pathway [31]. In the study, RSV and DGZ pretreatment upregulated the expression of p-PI3K, p-AKt, and p-mTOR compared with the H/R model group, suggesting that RSV and DGZ suppress apoptosis via the activation of the PI3K/AKt/mTOR signaling pathway.

## 5 Conclusion

RSV combined with DGZ had a synergistic cardioprotective effect against myocardium I/R injury through antagonizing apoptosis. The myocardial beneficial effects of RSV and DGZ were associated with the activation of the PI3K/AKt/mTOR pathway.
